# Neuropsychological and Neurophysiological Indicators of General and Food-Specific Impulsivity in Children with Overweight and Obesity: A Pilot Study

**DOI:** 10.3390/nu10121983

**Published:** 2018-12-15

**Authors:** Ricarda Schmidt, Caroline Sebert, Christine Kösling, Martin Grunwald, Anja Hilbert, Claudia Hübner, Lisa Schäfer

**Affiliations:** 1 Integrated Research and Treatment Center Adiposity Diseases, Department of Medical Psychology and Medical Sociology and Psychosomatic Medicine and Psychotherapy, Leipzig University Medical Center, 04103 Leipzig, Germany; Caroline.Sebert@medizin.uni-leipzig.de (C.S.); Christine.Koesling@medizin.uni-leipzig.de (C.K.); Anja.Hilbert@medizin.uni-leipzig.de (A.H.); Claudia.Huebner@medizin.uni-leipzig.de (C.H.); Lisa.Schaefer@medizin.uni-leipzig.de (L.S.); 2 Haptic-Research Laboratory, Paul-Flechsig-Institute for Brain Research, University of Leipzig, 04103 Leipzig, Germany; mgrun@medizin.uni-leipzig.de

**Keywords:** EEG, frequency bands, obesity, brain activity, impulsivity, children

## Abstract

Impulsivity, particularly towards food, is a potential risk factor for increased energy intake and the development and maintenance of obesity in children. However, neuropsychological and neurophysiological indicators of general and food-specific impulsivity and their association with children’s weight status are poorly understood. This pilot study examined electroencephalography (EEG) frequency band profiles during eyes-closed and eyes-open resting state in *n* = 12 children with overweight or obesity versus *n* = 22 normal-weight controls and their link to child- and parent-reported and experimentally assessed impulsivity of children (e.g., risk-taking behavior, approach-avoidance behavior towards food). The main results indicated that children with overweight/obesity versus normal weight showed significantly increased delta and decreased alpha band activity during eyes-closed resting state. Across the total sample, EEG slow-wave band activity was particularly linked to self- and parent-reported impulsivity and greater risk-taking behavior, but not to approach behavior towards food, after controlling for children’s age and weight status. The identification of specific EEG patterns in children with excess weight may provide a new basis for developing neurophysiological diagnostic and treatment approaches for childhood obesity. Future studies with larger samples and longitudinal designs are needed to replicate the present findings and test their stability over time.

## 1. Introduction

During the last decades, an increasing number of studies focused on the neuronal basis of obesity, mostly based on functional magnet resonance imaging or electroencephalography (EEG, for reviews see References [1,2,3]). Consistently, neuroimaging studies revealed altered neurophysiological processes in the orbitofrontal cortex [4,5,6], a region crucial for reward and reinforcement processes [7], and the dorsolateral prefrontal cortex, which is known to be involved in cognitive control [8,9,10], particularly in the processing of food cues [11]. Processes of both reward and cognitive control are major determinants of human approach and avoidance behaviors and can be termed as facets of impulsivity, defined as spontaneous, unplanned behavior with lack of regard or diminished sensitivity towards negative or long-term consequences [12]. In line with neuronal measures, neuropsychological measures of impulsivity indicated abnormalities in a number of facets of impulsivity in individuals with overweight and obesity across the age range compared to normal-weight controls [13].

In the general population, longitudinal studies indicated poor inhibitory control to predict higher body mass index (BMI, kg/m^2^) in children [14,15]. Recent research suggested that the association between impulsivity and weight status is driven by increased food intake [16,17]. In fact, experimental evidence revealed that children and adults with overweight and obesity were characterized by food-specific inhibition and interference deficits compared to normal-weight controls as measured by food-specific neuropsychological tasks (e.g., References [18,19,20,21,22]). However, while most evidence on impulsivity in children with obesity focused on the facets of inhibition or delay of gratification, there is little evidence on other dimensions of impulsivity, particularly decision-making, including risk-taking behavior [23] and automatic approach-avoidance tendencies towards food stimuli. Based on the Iowa Gambling Task (IGT) [24], an ecologically valid measure of decision-making under uncertainty in a context of punishment and reward, there is inconclusive evidence whether children and adolescents with excess weight show a general preference for disadvantageous, risky decisions compared to normal-weight controls [25,26] or not [27,28,29]. Similarly, studies applying other tasks than the IGT provided inconsistent findings on deviant decision-making in children and adolescents with excess versus normal weight [30,31]. As only two previous studies [25,30] used child-adapted tasks to examine decision-making in children and adolescents with excess weight, it is not clear whether methodological aspects accounted for the heterogeneity of findings.

Apart from behavioral measures, EEG profiles provide a more objective evaluation of pathological impulsivity across the age range [32]. The brain’s functional states as measured by resting-state EEG are relatively stable over time and may be used as an indicator of arousal, cognitive activation, and psychopathology [32,33]. Based on spectral power analysis, the EEG waveform can be classified within different bands spanning the frequency spectrum, ranging from slow-wave frequencies (delta, theta) to moderate (alpha) and high frequency activity (beta). A vast number of studies found increased slow-wave EEG activity to be associated with behavioral impulsivity, including reduced inhibition [34] and increased risk-taking behavior in adults with attention-deficit/hyperactivity disorder (ADHD) or healthy university students [35,36,37]. Clinically, children with spectral elevations of resting-state slow-wave frequencies, mainly theta, and/or reduced alpha and beta activity were likely to have a diagnosis of externalizing behavior, including ADHD or antisocial symptomatology [34,38,39], which are common comorbidities in childhood obesity [40]. In contrast, the preponderance of fast-wave activity during resting-state EEG was found to be related to slower reaction times during a neuropsychological task in adults with ADHD [41]. In a sample of adults with a gambling disorder, frontal and central alpha band activity during eyes-closed resting state was negatively associated with self-reported impulsivity [42]. Thus, conditions characterized by increased impulsivity were found to show an EEG profile with increased slow-wave and decreased fast-wave band activity, suggesting cortical hypoarousal or a maturational lag in these individuals, respectively.

Despite the evidence that resting-state EEG brain activity is a marker of behavioral impulsivity, EEG studies in children did not explicitly consider child weight status so far, leaving the question whether the neurophysiological profile in children with obesity is actually deviant. EEG studies focusing on early, event-related information processing in children and adolescents with overweight and obesity versus normal weight found general and food-specific reductions in inhibitory processing during task performance [43,44], but nothing is known about the continuous brain activity at rest. Following the assumption of a proposed neuronal overlap between childhood obesity and ADHD [45], the EEG profile of children with obesity should be expected to resemble that of children with ADHD, at least to a certain extent. The only two studies analyzing EEG spectral power in relation to child’s weight status revealed increased frontal beta in adolescent girls with overweight or obesity during a working memory task [46] and increased functional connectivity in the delta and beta band at eyes-closed rest [47] compared to normal-weight peers, but nothing is known about EEG spectral power at resting-state activity. Simultaneously, there is very little evidence on the EEG power spectrum and its associations with impulsivity in adults with obesity available [3]. Based on evidence from only two studies on the sources of resting-state EEG, adults with overweight and obesity showed lower parieto-occipital alpha oscillations during eyes-closed resting-state EEG [48], as well as posterior alpha desynchronization during eyes-open resting state compared to normal-weight adults [49] reflecting abnormal arousal/vigilance processes in these individuals.

Thus, the present pilot study sought to examine neuropsychological and neurophysiological indicators of impulsivity and their relation in children with overweight and obesity versus normal-weight controls. Specifically, this study aimed at investigating associations between eyes-closed and eyes-open resting-state EEG indices and neuropsychological, as well as questionnaire-based measures of general and food-specific impulsivity in children aged 8 to 13 years for the first time. Given the exploratory design of this study and the few available findings on EEG resting state activity in individuals with overweight and obesity, especially in children, both eyes-closed and eyes-open resting state were examined. It was hypothesized that children with overweight or obesity will exhibit greater slow-wave EEG band activity and lower fast-wave band activity during resting state than children with normal weight. According to previous findings in adults with ADHD [41] and the hypoarousal model of ADHD [50], it was expected that group differences would be more pronounced during eyes-closed resting-state, assuming that children with overweight and obesity are cortically under-aroused during eyes-closed resting condition and seeking for external stimulation. In order to assess children’s impulsivity as detailed as possible and to reduce age-related biases in the context of self-report questionnaires, parent-report measures were used as well. Since EEG frequency band activity was evidenced to be a general indicator of impulsivity both in clinical child and non-clinical adult samples [36,41], the study aimed to evaluate the association between EEG resting-state activity and multiple indicators of impulsivity in a non-clinical child sample using the total sample. Specifically, it was expected that EEG resting-state slow-wave band activity would be positively and fast-wave band activity would be negatively related to experimentally assessed decision-making and automatic approach-avoidance tendencies towards foods, as well as self- and parent-reported general and food-specific impulsivity, respectively.

## 2. Materials and Methods

### 2.1. Participants

Eligible participants were German-speaking children aged 8 to 13 years with overweight or obesity, and normal weight, respectively, who were recruited from the population (e.g., via advertisements on the Internet, supermarkets) and clinical institutions (e.g., University Medical Center, outpatient weight reduction programs). Inclusion in the experimental group (EG) required objectively measured overweight or obesity based on age- and sex-specific German reference data [51]. Overweight and obesity were defined via BMI-standard deviation scores (SDS) ≥ 1.28 and ≥ 1.88, respectively. Children were assigned to the control group (CG) if they had normal weight (−1.28 ≤ BMI-SDS < 1.28). Exclusion criteria for both groups included parent-reported left-handedness, mental, neurological, or serious physical disorders (e.g., ADHD), medication intake affecting executive functions or brain activity, or mental retardation of the child. The pilot study was approved by the local Ethics Committee of the University of Leipzig.

The EG was comprised of *n* = 5 children with overweight and *n* = 7 children with obesity. The CG consisted of *n* = 22 children with normal weight, stratified to the EG for age and sex. By design, the groups significantly differed in their weight status, with the EG having a higher BMI-SDS than the CG (*p* < 0.001), but also in their socioeconomic status [52], with children of the EG having a lower socioeconomic status (*p* < 0.01) than the CG (Table 1).

### 2.2. Procedure

The standardized experimental sessions were conducted by two highly trained PhD and MD students. In the beginning, written informed consent and assent were obtained from the parent and child, respectively, followed by children’s intelligence assessment. During the subsequent EEG preparation, children completed self-report questionnaires, while parents were asked to do so at home beforehand. After EEG recordings and a 5-min break, neuropsychological tests were conducted. Subsequently, children’s eating disorder psychopathology and mental comorbidities were assessed via separate clinical interviews with the child and one parent, respectively (Eating Disorder Examination adapted for Children [53,54], K-DIPS [55]). The experimental session ended with the objective assessment of children’s weight and height. Altogether, the experimental session took approximately 3 h. Children and their parents received a financial compensation of 15€ for participation.

### 2.3. EEG Recording and Analysis

EEG recordings took place in an acoustically and electromagnetically shielded cabin with video control and were conducted using a 32 channel QuickAmp amplifier and Brain Vision Analyzer 2.1 software (Brain Products, Gilching, Germany). For all recordings, participants were grounded peripherally following the standard 10–20 International system with linked ear referencing to obtain EEG activity from 19 scalp sites (Fp1, Fp2, F3, F4, F7, F8, Fz, C3, C4, Cz, T3, T4, T5, T6, P3, P4, Pz, O1, and O2) at a sampling rate of 250 Hz. Eye movements and blinks were measured with two bipolar EOG electrodes placed 1 cm beyond the outer edge of both eyes and 1 cm below and above the right eye. EEG resting state was recorded for 300 s with eyes-open (EO) while looking at a fixation cross on the display and for 300 s with eyes-closed (EC). The order of EEG resting state with EO and EC was randomized.

A band-pass filter of 0.53–70 Hz and a notch filter of 50 Hz were used to filter EEG data. The continuous EEG was segmented in 2-s intervals and ocular artifact correction was conducted in accordance with Gratton et al. [56]. Further, automatic artifact rejection was applied to segments with voltage steps greater than 50 µV/ms and amplitudes exceeding ±100 µV. Subsequent to these automated procedures, two extensively trained researchers visually scanned all data in order to identify and remove residual contaminants (e.g., artifacts, focal abnormalities, drowsiness). At least 30 artifact-free segments (not necessarily consecutive) of the filtered EEG data were required for EO and EC to be included in the analysis. As a consequence, *n* = 10 children of the EG (*Mean [M]* = 57.8 segments; *Standard Deviation [SD]* = 19.8; range 36–88) and *n* = 19 children of the CG (*M* = 78.32 segments; *SD* = 28.1; range 39–127) were included in further analyses in the EO condition. Regarding EC, *n* = 11 children of the EG (*M* = 108.3 segments; *SD* = 38.0; range 41–150) and *n* = 22 children of the CG (*M* = 89.4 segments; *SD* = 30.9; range 40–156) were included in the subsequent analyses. The filtered EEG data were Fourier transformed with a Hanning window length of 20% extracting delta (δ, 1.0–3.5 Hz), theta (θ, 3.5–7.5 Hz), alpha (α, 7.5–12.5 Hz), and beta (β, 12.5–30 Hz) frequency bands. The extracted absolute power for each frequency band was grouped for frontal (Fp1, Fp2, F3, F4, F7, F8, Fz), central (C3, C4, Cz), temporal (T3, T4, T5, T6), parietal (P3, P4, Pz), and occipital (O1, O2) regions, converted to relative band power (%), and ln-transformed before statistical hypotheses testing to obtain normally distributed data.

### 2.4. Neuropsychological Tasks

Approach-Avoidance Task (AAT). The study-specific AAT was adapted from Wiers et al. [57] and is a computerized measure for assessing approach and avoidance tendencies towards food in children. Following the principle of the AAT, children had to move the computer mouse as quickly and as accurately as possible in dependence of the format of pictures presented on a computer screen (portrait or landscape): By pulling the computer mouse to the bottom of the computer screen, the picture grows bigger (approach), while by pushing the computer mouse to the top of the screen, the picture size decreases (avoidance). After successful completion of a test run, including 10 grey rectangles, 40 pictures were presented in both landscape and portrait format in a randomized order, summing up to 80 trials. Specifically, there are four picture categories with 10 pictures each presenting high-calorie foods (e.g., burger), low-calorie foods (e.g., banana), pleasant neutral stimuli (e.g., smartphone), as well as boring neutral stimuli (e.g., ladder) to evaluate automatic responses depending on stimulus content. Boring neutral pictures and food pictures were pre-selected from a large, validated picture data base [58] based on the highest ratings of perceptibility and high familiarity. Finally, food pictures and pleasant neutral pictures were selected based on the criteria of high liking and perceptibility derived from a previous independent rating of food and pleasant neutral pictures in 38 children of two elementary schools aged between 8 and 12 years. In accordance to Wiers et al. [57], one child with overweight and two children with normal weight had to be excluded based on error percentages greater than 25%. Per each picture category, difference scores were computed based on mean reaction times in ms for the onset of correct responses to approach and avoidance trials, for example, “high calorie food/push—high calorie food/pull.” Positive scores indicate relatively faster reaction times for approach responses compared to avoidance, hence an approach behavior. In the present study, only approach-avoidance tendencies for low-calorie and high-calorie food pictures were analyzed.

Youth Version of the Balloon Analogue Risk Task (BART-Y). The BART-Y [59] is a computerized task for the assessment of risk-taking behavior through balancing reward versus loss of points. Children were instructed to pump up 30 computer-generated balloons by clicking a button. With each click, the balloon was inflated further, and points were added to a counter up to a certain threshold at which the balloon explodes. If the child decided to cash-out before the balloon explodes, the accumulated points for that trial were added to the counter. However, if the balloon exploded, the child lost the points earned so far for that trial. Children were not informed about the balloons’ break point that varied across each of the 30 trials. Thus, each pump confers greater risk, but also greater potential reward. After balloon explosion or saving of points, a new balloon appeared. At the end of the task, collected points were exchanged to differently sized prizes (small, medium, large, and bonus toy). Risk-taking behavior was measured by the average number of pumps on balloons that did not explode, with higher scores indicating higher risk-taking behavior.

### 2.5. Self-Report Questionnaires

To assess children’s eating behavior and food-specific approach-behaviors, the subscales ‘enjoyment of food’ (α = Cronbach’s α in this study’s sample = 0.81) and ‘food responsiveness’ (α = 0.91) of the Children’s Eating Behavior Questionnaire (CEBQ) [60] were administrated to parents and mean scores were computed for each subscale. Sum scores of the subscale ‘hyperactivity’ from the parent version of the Strength and Difficulties Questionnaire (SDQ; α = 0.72, [61]) were used to determine children’s general impulsivity. Additionally, children’s self-reported general impulsivity was measured using the sum score of the subscale ‘impulsivity’ of the Inventory for the Assessment of Impulsivity, Venturesomeness and Empathy in 9–14 years old children (IVE, α = 0.62, [62]) which is an adapted German version of the Impulsivity Questionnaire I6 by Eysenck and Eysenck [63].

### 2.6. Intelligence

Children’s intelligence was estimated using the ‘matrix reasoning subtest’ of the Wechsler Intelligence Scale for Children—Fourth edition (WISC-IV; [64,65]) which nonverbally assesses abstract-logical reasoning. Subtest scores were converted into age-specific standard values ranging between 1 and 19, with higher scores indicating higher levels of intelligence.

### 2.7. Data Analytic Plan

Statistical analyses were performed using IBM SPSS Statistics version 23 (IBM Corp. Released 2015. IBM SPSS Statistics for Windows, Version 23.0. Armonk, NY: IBM Corp) and consisted of two steps. First, group differences between the EG and CG regarding impulsivity measures and resting-state EEG activity measured during EC and EO were examined using two-tailed independent sample *t* tests. In order to correct for possible inflations of the Type I error rate due to multiple testing, an adjusted significance level of *p* < 0.05/20 = 0.0025 was considered to indicate statistical significance. In terms of violation of normality and homogeneity of variances, non-parametric tests were used and reported if results differed from the parametric tests. Effect sizes of group differences were reported by Cohen’s *d*, whereby values ≥ 0.20 refer to small, ≥ 0.50 to medium, and ≥ 0.80 to large effects [66].

Second, associations between children’s EEG activity and various impulsivity indicators (AAT, BART-Y, CEBQ, SDQ, IVE) over and above group status (EG vs. CG) were examined via partial correlations (Pearson) with children’s weight status (BMI-SDS) and age serving as control variables to exclude weight- and development-related effects on measured EEG activity [67]. Effect sizes of correlations were interpreted according to Cohen with values *r* ≥ 0.10 referring to small, *r* ≥ 0.30 to medium, and *r* ≥ 0.50 to large effects [66]. Statistical significance for correlation analyses was set at α < 0.05. Post-hoc power analyses revealed adequate power (1 − β = 0.80) for detecting large-sized group differences (*d* > 1) in EEG resting state and medium-to-large-sized effects (*r* > 0.40) in correlation analyses.

## 3. Results

### 3.1. Neuropsychological Tasks

While the EG showed an avoidance behavior and the CG an approach behavior of medium effect size regarding high-calorie foods, an approach behavior to low-calorie foods was found in both groups; however, biases were not significantly different from 0 (*p* > 0.05). The EG and CG did not significantly differ in approach or avoidance behaviors to high- or low-calorie foods in the AAT (*p* > 0.05, Table 2).

A non-significant (*p* > 0.05), but medium-sized group difference was found for the number of pumps in the BART-Y, with the EG presenting a lower average number of pumps than the CG, indicating lower risk-taking behavior.

### 3.2. Self-Report Questionnaires

Parents of children of the EG reported significantly higher levels of ‘food responsiveness’ (*p* < 0.01, large effect) and marginally significantly higher levels on the subscale ‘enjoyment of food’ (*p* < 0.100, medium effect) of the CEBQ compared to the CG.

For general self- and parent-reported impulsivity, small-to-medium-sized, but non-significant group differences were revealed (*p* > 0.05) indicating descriptively higher levels of impulsivity in the EG than the CG.

### 3.3. Resting-State EEG Activity

As depicted in Table 3, the EG showed significantly higher delta band activity than the CG during EC resting state with large-sized effects for parietal and occipital regions (*p* ≤ 0.003), while greater delta band activity in the EG versus CG did not reach significance for other regions (0.022 ≤ *p*s ≤ 0.149). Although non-significant, large-sized group effects were found for greater occipital theta band activity and lower alpha band activity in all brain regions in the EG versus CG (0.007 ≤ *p*s ≤ 0.036). Descriptively higher theta and beta band activity in the EG versus CG did not reach significance (*p*s ≥ 0.027).

For resting-state EEG activity measured during EO, no significant group differences were found for any frequency band and any brain region (0.333 ≤ *p*s ≤ 0.998).

### 3.4. Associations between Resting-State EEG Activity and Impulsivity Measures

**Figure 1:**
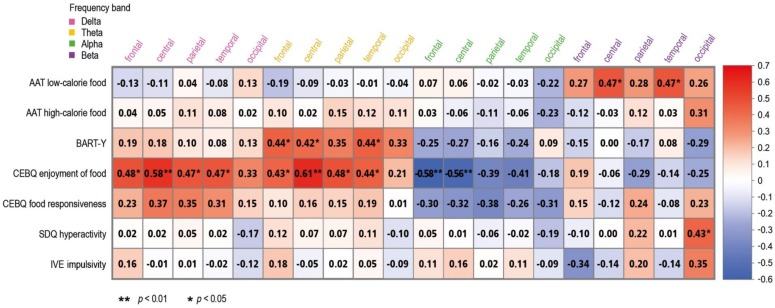
Matrix of correlations between eyes-open EEG resting-state activity, Approach-Avoidance-Task (AAT), the Youth Version of the Balloon Analogue Risk Task (BART-Y), parent-reported Child Eating Behavior Questionnaire (CEBQ), Strengths and Difficulties Questionnaire (SDQ), and child-reported Inventory of Venturesomeness and Empathy in 9–14 years old children (IVE) across the total sample with valid EEG data (*n* = 29), while controlling for child age and weight status.

Eyes-open. Results of the correlation analyses between frequency band activities measured during EO resting-state EEG and neuropsychological indices of impulsivity are presented in Figure 1. After controlling for children’s age and weight status, increased slow-wave band activity (i.e., delta and theta) found in frontal, central, parietal, and temporal regions was significantly associated with higher scores in the CEBQ subscale ‘enjoyment of food’ with medium-to-large-sized effects (0.002 ≤ *p*s ≤ 0.043). Additionally, significant positive correlations of medium effect size were detected between frontal, central, and temporal theta band activity and average numbers of pumps in the BART-Y (0.038 ≤ *p*s ≤ 0.046).

Regarding fast-wave band activity (i.e., alpha and beta), increased frontal and central alpha band activity was significantly associated with lower scores in the CEBQ subscale ‘enjoyment of food’ with medium-to-large-sized effects (0.004 ≤ *p*s ≤ 0.005). Increased central and temporal beta band activity was significantly associated with approach behavior for low-calorie food in the AAT with medium-to-large-sized effects (0.023 ≤ *p*s ≤ 0.024). Medium-sized significant positive correlations were revealed between occipital beta band activity and the SDQ subscale ‘hyperactivity’ (*p* = 0.044). No other significant correlations were found during EO.

Eyes-closed. After controlling for children’s age and weight status, occipital delta band activity and average numbers of pumps in the BART-Y were negatively correlated with medium-sized effects (*p* = 0.028).

Regarding fast-wave band activity, increased beta band activity in frontal regions was associated with lower scores on the IVE subscale ‘impulsivity’ (*p* = 0.021, medium-to-large-sized effect). Other correlations between EC resting-state EEG and impulsivity indices were not significant.

## 4. Discussion

To our knowledge, this is the first study that investigated both eyes-closed (EC) and eyes-open (EO) resting-state EEG activity in 8-to-13-year-old children with overweight and obesity versus normal weight and its associations with general and food-specific impulsivity measured experimentally and via self- and parent-report. The results revealed an obesity-specific EEG profile characterized by greater slow-wave and decreased alpha band activity during EC resting-state EEG, congruent with EEG patterns found in other conditions marked by increased impulsivity, such as ADHD (e.g., References [68,69]). Independent of children’s weight status, increased slow-wave and decreased alpha band activity during EO resting state were associated with higher impulsivity, characterized by greater risk-taking behavior during a neuropsychological task and parent-reported food-approach behaviors, mirroring findings in youth with ADHD [70] and healthy individuals [35,36].

The preponderance of slow-wave band activity and reductions of alpha band activity during EC resting state in children with overweight and obesity suggested neuronal similarities with ADHD, a highly comorbid disorder to obesity across the age range [40]. Because a concurrent diagnosis of ADHD was an exclusion criterion for the present sample, the results were not contaminated by underlying neuronal abnormalities due to the presence of this neurodevelopmental disorder. Notably, disorder-specific symptoms in individuals with deviant EEG patterns are considered to reflect compensatory mechanisms to counter-regulate the vigilance level, either via withdrawal and sensation avoidance (depression) or inattention, hyperactivity/impulsivity [71]. Thus, general deficiencies in self-control in children with obesity might be attributable to cortical hypoarousal, as in those with ADHD or other externalizing behavior [39]. Although there are no studies available addressing the particular aspect of the association between cortical arousal and self-regulation neither in adults nor in children with overweight and obesity, preliminary evidence in adults with obesity indicated that stimulant treatment, such as methylphenidate, may have an effect on the reduction of food intake, at least in some individuals [72] proposing that there might be an EEG phenotype of slow-wave preponderance in individuals with obesity. According to Knyazev (2012) [73], delta band activity represents the most basic evolutionary old oscillatory mode in animals and humans and is especially associated with motivational processes and biological rewards, including food, suggesting that children with overweight and obesity may be neurophysiologically characterized by persistent states of attention to motivationally rewarding stimuli.

Existing evidence on resting-state EEG activity in individuals with obesity was based on two studies in adults with obesity and comorbid binge-eating disorder (BED) [74,75]. Consistently, these studies revealed increased beta band activity and functional connectivity, respectively, in adults with obesity with versus without BED, while there were no group differences for other frequency bands [74,75]. However, the present study did not reveal significant differences in the beta band in either resting-state condition between children with overweight and obesity versus normal weight suggesting that increased beta band activity has a crucial role for individuals with obesity and high levels of food-specific impulsivity characterizing those with comorbid BED, but not children with obesity only. Notably, none of these previous studies on EEG resting-state activity included a control group of normal-weight individuals so far, making specific comparisons to these previous studies difficult [74,75]. It therefore remains to be further examined whether there are EEG subtypes in individuals with obesity and/or BED, possibly reflecting the heterogeneity in eating disorder and general psychopathology, including impulsivity in this population [76,77], in accordance with findings in ADHD research [78,79].

The fact that children with overweight and obesity versus those with normal weight did not show greater approach behavior towards high-calorie foods during the AAT is contrary to expectations, but fits into the inconsistent findings of previous studies in child and adult samples (e.g., References [80,81]). Within an intervention study in 276 8–16 years old youth, baseline AAT data already indicated avoidance tendencies for snack foods in children with obesity, much like in the present study; however, there were no data of a normal-weight control group available [82]. Similarly, the only study investigating adults with obesity did not find an approach bias towards high- or low-calorie foods [81], while university students with high levels of food cravings showed greater approach behavior towards food cues than adults with low levels of food craving [83]. The lack of effects may mirror conditioned responses towards high-calorie foods, the anticipation of punishment, or repeated reminders to avoid high-calorie foods, because children with obesity were mainly recruited via weight loss facilities. As the AAT is a reaction time task, socially desirable response behavior can be ruled out. In contrast, the BART-Y does not include any time component, but reflexive decision-making. The results of the BART-Y were unexpected as well, because children with overweight and obesity were not found to decide highly risky and did not strive for a large reward. Thus, the present results add to the inconsistent evidence on decision-making and risk-taking behavior in children with obesity (e.g., References [25,28]). Compared to the IGT or its age-adapted versions, such as the Hungry Donkey Task [25], the component of punishment in the BART-Y is more emotionally intensive as it is presented by an annoying tone when the balloon explodes. Thus, the results obtained from the BART-Y may rather mirror sensitivity to punishment or increased levels of anxiety (e.g., Reference [84]) than risk-taking behavior in children with obesity. Importantly, the questionnaire-based measures indicated higher levels of food-specific and general impulsivity in children with overweight and obesity versus normal-weight controls, consistent with recent findings [85].

Given that EEG resting-state frequency band activity serves as an electrophysiological signature for a range of impulsivity facets [32], correlational analyses between these measures were conducted across the total sample of children with normal weight, overweight, and obesity. In line with expectations and previous findings [34,35,36,37], delta and theta band activity during EO was positively related to parent-reported levels of enjoyment of food, describing strong approach behaviors towards foods. In contrast, but as expected [42], the direction of the association between alpha band activity and parent-reported food approach tendencies was the opposite (i.e., negative). After controlling for children’s weight status and age, children’s theta band activity and risk-taking behavior in the BART-Y were positively associated, as found in previous studies using the IGT in university student samples [35,36,37]. Notably, these associations were consistently found across many brain regions underlying the strength and connectivity of findings. For fast-wave band activity, positive relations between occipital beta band activity and children’s level of hyperactivity were revealed, which goes in line with studies in adult and adolescent samples with obesity, although these findings were particularly relevant for frontal brain regions (for a review, see Reference [3]).

Compared to the associations found between EO resting-state frequency band activity and impulsivity measures the results on EC resting-state correlates were more selective regarding brain region and the direction of the association. For example, occipital delta band activity was negatively associated with risk-taking decisions during the BART-Y. These localized changes in the direction and topography of effects might be related to task-specific activation processes or the differential cortical processing of visual input, as EO and EC conditions generally produce differences in power and topography levels in children and adults [86,87].

A major strength of this pilot study is the standardized record of children’s resting-state EEG activity in an acoustically and electromagnetically shielded cabin with video control. To increase the validity of the present results, EEG data were cleaned from artifacts by two independent experts. Furthermore, behavioral parameters of impulsivity were multimodally assessed via neuropsychological tasks with child-adapted food-specific and neutral stimuli and state-of-the-art questionnaires. For the first time, children’s weight status was considered to describe specific EEG patterns in children with overweight and obesity under exclusion of ADHD and eating disorders.

However, some limitations must be considered when interpreting the results. Only large effects could be detected with sufficient power, due to the small sample size. For this reason, non-parametric tests were used where necessary and the corresponding effect sizes were reported. In addition to children with obesity, children with overweight were included in the EG, possibly attenuating between-group differences in EEG profiles related to obesity only. Due to the cross-sectional design, no conclusions can be drawn about the predictive value of EEG profiles on children’s eating behavior and weight status.

Clinically, the identification of EEG profiles specific to childhood overweight and obesity may allow for taking a neurophysiological approach to target insufficient self-regulation in obesity, for example via EEG neurofeedback. Recent studies revealed promising effects of a specific EEG frequency band training for reducing the number of binge-eating episodes in women with obesity [88]. From a research point of view, this study implicates the need for further EEG studies in childhood obesity using larger sample sizes for detecting small-to-medium-sized group differences and longitudinal designs to evaluate the stability of EEG profiles over time. In this context, future studies should consider using alternative measures of impulsivity to cover its multifaceted nature, for example, via the stop signal task [89], a measure of response inhibition. Finally, research is warranted to determine the presence of EEG subtypes in children with obesity in order to account for the expected neurophysiological heterogeneity similar to that in ADHD (e.g., Reference [90]).

## Figures and Tables

**Table 1:** Sociodemographic description of the experimental group (EG) and control group (CG).

	EG (*n* = 12)	CG (*n* = 22)			
	*n (%)*	*n (%)*	χ^2^	*df*	*p*
Sociodemographics					
Sex (female)	8 (66.7)	10 (45.5)	1.40	1	0.236
	*M (SD)*	*M (SD)*	*t*	*df*	*p*
Age (years)	10.9 (1.9)	10.1 (1.6)	1.26	1, 32	0.217
BMI percentile (0–100)	96.5 (3.3)	54.8 (20.7)	9.25	1, 22.9	<0.001
BMI-SDS	2.09 (0.49)	0.14 (0.60)	9.60	1, 32	<0.001
SES Winkler index (0–21)	12.3 (3.8)	15.8 (3.2)	−2.82	1, 32	0.008
Intelligence					
Matrix reasoning (1–19)	11.2 (2.8)	12.6 (1.8)	−1.55	1, 16.1	0.141

Note: BMI body mass index; CG control group; EG experimental group; *M* mean; *SD* standard deviation; SDS standard deviation score; SES socioeconomic status.

**Table 2:** Self-reported and neuropsychological measures of general and food-specific impulsivity as a function of weight status.

	EG	CG				
Neuropsychological Tasks	*M (SD)*	*M (SD)*	*t*	*df*	*p*	*d*
AAT (ms)						
High-calorie food	−12.2 (134.5)	70.6 (171.3)	−1.38	1, 29	0.177	−0.52
Low-calorie food	39.2 (272.1)	82.2 (230.6)	−0.46	1, 28	0.649	−0.18
BART-Y						
*N* pumps	17.9 (9.0)	24.0 (10.8)	−1.66	1, 31	0.108	−0.60
**Self-report questionnaires**	***M (SD)***	***M (SD)***	***t***	***df***	***p***	***d***
CEBQ						
Enjoyment of Food	3.2 (0.7)	2.9 (0.5)	1.79	1, 32	0.083	0.52
Food Responsiveness	2.2 (1.2)	0.9 (0.5)	3.54	1, 13.2	0.004	1.60
IVE						
Impulsivity	8.1 (3.2)	6.7 (2.8)	1.35	1, 32	0.188	0.48
SDQ						
Hyperactivity	3.3 (2.1)	2.6 (1.9)	0.94	1, 32	0.357	0.36

Note. AAT Approach Avoidance Task; BART-Y Youth Version of the Balloon Analogue Risk Task; CEBQ Children’s Eating Behavior Questionnaire; CG control group; EG experimental group; IVE Inventory for the Assessment of Impulsivity, Venturesomeness and Empathy in 9–14 years old children; *M* mean; *SD* standard deviation; SDQ Strengths and Difficulties Questionnaire. Negative *d* values indicate that the EG scored lower in the respective measure than the CG.

**Table 3:** Eyes-closed resting-state electroencephalography (EEG) activity as a function of weight status.

	EG	CG				
	*M (SD)*	*M (SD)*	*t*	*df*	*p*	*d*
Delta (µV^2^)						
Frontal	−1.07 (0.18)	−1.18 (0.21)	1.48	1, 31	0.149	0.55
Central	−1.19 (0.15)	−1.34 (0.19)	2.41	1, 31	0.022	0.84
Parietal	−1.25 (0.19)	−1.51 (0.23)	3.26	1, 31	0.003	1.19
Temporal	−1.22 (0.17)	−1.35 (0.17)	2.13	1, 31	0.041	0.77
Occipital	−1.29 (0.23)	−1.71 (0.38)	3.31	1, 31	0.002	1.24
Theta (µV^2^)						
Frontal	−1.27 (0.16)	−1.37 (0.26)	1.21	1, 31	0.235	0.43
Central	−1.16 (0.12)	−1.30 (0.25)	1.84	1, 31	0.975	0.65
Parietal	−1.31 (0.15)	−1.51 (0.32)	1.96	1, 31	0.059	0.72
Temporal	−1.23 (0.13)	−1.38 (0.24)	1.88	1, 31	0.069	0.71
Occipital	−1.48 (0.23)	−1.83 (0.46)	2.33	1, 31	0.027	0.87
Alpha (µV^2^)						
Frontal	−1.54 (0.32)	−1.26 (0.34)	−2.29	1, 31	0.029	−0.84
Central	−1.41 (0.34)	−1.06 (0.36)	−2.73	1, 31	0.010	−0.99
Parietal	−1.26 (0.30)	−0.94 (0.30)	−2.88	1, 31	0.007	−1.07
Temporal	−1.33 (0.31)	−1.09 (0.30)	−2.19	1, 31	0.036	−0.79
Occipital	−1.00 (0.33)	−0.69 (0.36)	−2.36	1, 31	0.025	−0.88
Beta (µV^2^)						
Frontal	−1.95 (0.22)	−2.11 (0.34)	1.41	1, 31	0.167	0.52
Central	−2.06 (0.12)	−2.19 (0.34)	1.27	1, 31	0.214	0.45
Parietal	−2.25 (0.26)	−2.36 (0.33)	0.93	1, 31	0.360	0.36
Temporal	−2.05 (0.19)	−2.12 (0.34)	0.62	1, 31	0.538	0.23
Occipital	−2.37 (0.37)	−2.43 (0.43)	0.39	1, 31	0.703	0.15

Note: Percentage EEG activity at a particular location increases as the mean of the ln-transformed data becomes less negative. Negative *d* values indicate that the EG scored lower in the respective measure than the CG. *M* mean; *SD* standard deviation.
